# Optimization of solvent and extraction time on secondary metabolite content of mangosteen leaf (*Garcinia mangostana* L.) as a feed additive candidate on poultry

**DOI:** 10.5455/javar.2024.k758

**Published:** 2024-03-31

**Authors:** Ridho Kurniawan Rusli, Maria Endo Mahata, Ahadiyah Yuniza, Zurmiati Zurmiati, Sepri Reski, Cecep Hidayat, Mustofa Hilmi, Rita Mutia

**Affiliations:** 1 Department of Nutrition and Feed Technology, Faculty of Animal Husbandry, Universitas Andalas, Padang, Indonesia; 2 The National Research and Innovation Agency, Jakarta, Indonesia; 3 Study Program of Livestock Product Processing Technology, Politeknik Negeri Banyuwangi, Banyuwangi, Indonesia; 4 Department of Nutrition and Feed Technology, Faculty of Animal Science, IPB University, Bogor, Indonesia

**Keywords:** Extraction, Feed additive, Mangosteen leaf, Phytochemical components

## Abstract

**Objectives:**

This research aimed to determine the optimum type of solvent and extraction time to produce secondary metabolites (phenolics, flavonoids, tannins, and antioxidants) from mangosteen leaves (*Garcinia mangostana* L.) as feed additive candidates for poultry.

**Materials and Methods:**

This research used a completely randomized design with a 2 × 5 factorial design with three replications. Factor A used two types of distilled water as a solvent (ordinary distilled water and distilled water heated at 100°C), while Factor B encompassed various extraction times (15, 30, 45, 60, and 75 min). The parameters assessed included total phenolic content (TPC), total flavonoid content (TFC), total tannin content (TTC), and overall antioxidant activity.

**Results:**

The TPC, TFC, TTC, and total antioxidant activity all showed a highly significant interaction (*p* < 0.01) with the type of solvent and extraction duration.

**Conclusion:**

The best solvent and time for mangosteen leaf extract to produce secondary metabolites, which can be candidates for feed additives in poultry, is ordinary distilled water for 45 min. In this research, the phenol content was 81.03%, flavonoids 11.07%, tannins 1.01%, and antioxidants 77.61%.

## Introduction

The poultry population in modern and developing countries has witnessed a consistent year-to-year increase, primarily attributed to significant advancements in genetics. This development cannot be separated from the rapid progress in genetics so that poultry (especially broilers and laying hens) can be produced relatively quickly and efficiently. So far, antibiotic growth promoters (AGPs) are often used to increase poultry productivity. However, starting in 2018, in Indonesia, the utilization of AGP as a supplement in animal feed has been prohibited because of concerns about its potential influence on bacterial resistance and chemical residues in poultry products consumed by consumers. Consequently, banning AGP in poultry feed has impacted decreasing chicken production and health. In recent years, there has been research exploring the application of natural growth promoters in poultry farming, such as green algae [1], propolis [2], oregano [3], graviola [4], cinnamon, and onion [5].

Indonesia boasts a potentially valuable natural growth promoter in mangosteen (*Garcinia mangostana L.*). The mangosteen rind (powder and extract) is rich in several bioactive compounds, including xanthones and their derivatives [6], anthocyanins [7], phenolic compounds, flavonoids, and alkaloids [8]. These active compounds have properties such as antioxidants [6, 8], anti-bacterial, anti-proliferative, anti-inflammatory [6], and anti-cancer [7]. These compounds effectively increase appetite, immunity, digestive tract health, and antioxidants, improving livestock performance. Mangosteen peel can be given in extract form to broilers [9], quail [10], and meal to laying hens [11,12].

In addition to mangosteen rind, mangosteen leaves contain active compounds such as flavonoids, alkaloids, saponins, and tannins [13]. Some research results show that extracting mangosteen leaves can use ethanol [13,14] and methanol [15], but these solvents are not applicable and expensive when applied to the livestock sector. Information regarding the use of distilled water and extraction time to produce secondary metabolites is still limited. In this context, this study was designed. The main objective was to validate mangosteen leaf extract as a source of phytochemical compounds used as feed additive candidates in poultry. Specifically, the objective of this research was to extract and assess bioactive compounds from mangosteen leaves using various extraction times and solvents.

## Material and Methods

### Materials

Mangosteen leaves (*Garcinia mangostana* L.) were from a local plantation in Koto Lua Village, Padang City, West Sumatra, Indonesia.

### Preparation of extract

**Figure 1.**
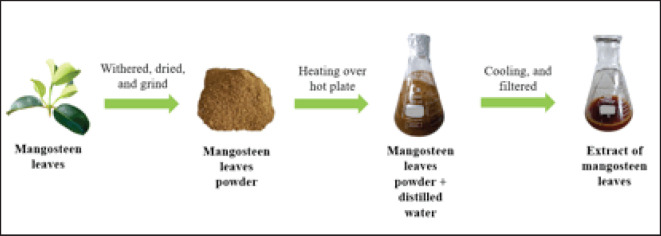
Procedure extract of mangosteen leaf.

The mangosteen leaf extraction process (Fig. 1) refers to Yassin et al. [16] with some modifications. Mangosteen leaves (shoots) are washed thoroughly with running water to remove dust and other impurities. After that, the mangosteen leaves were withered for 24 h, then dried in an oven at 50°C for 24 h. After drying, the mangosteen leaves were ground and then filtered (355 µm, Endecotts Ltd., London, England). 10 gm of mangosteen leaf powder is mixed with 100 ml of distilled water, both ordinary and heated distilled water. Furthermore, the extraction process was carried out using a hot plate with a processing time of 15, 30, 45, 60, and 75 min at 50°C. After that, the extracted mangosteen leaves were cooled at room temperature and then filtered twice using Whatman paper No. 1 (Cytiva, China). The mangosteen leaf extraction results were stored at a refrigerator temperature of 4°C.

### Experimental design

This research used a completely randomized design with a 2 × 5 factorial design with three replications. Factor A, two types of distilled water as a solvent (ordinary distilled water and distilled water heated at 100°C), and factor B, different extraction times (15, 30, 45, 60, and 75 min).

### Total phenolic content (TPC)

Analysis of TPC Using the Folin-Ciocalteu method by Calvindi et al. [17], In a microplate, up to 20 µl of sample extract and 120 µl of 10% (v/v) Folin-Ciocalteu reagent were combined. Next, let it sit at room temperature for 5 min. 80 µl of a 7.5% Na_2_CO_3_ solution was added to the mixture after incubation. After that, the mixture was left to stand at room temperature in the dark for 30 min. The sample’s absorbance was then determined using a spectrophotometer (Shimadzu UV-1800, Japan) at a wavelength of 725 nm. The measurement for TPC is mg gallic acid equivalent (GAE)/gm dry weight.

### Total flavonoid content (TFC)

TFC analysis was based on the procedure performed by Chang et al. [18]. The sample is diluted beforehand with a ratio between the sample (1 gm) and methanol (10 ml). Then 1 ml of the sample was added with 3 ml of methanol, 0.2 ml of 2% ALCL_3_, 0.2 ml of 1 M glacial acetic acid, and 5.6 ml of distilled water. Then the mixture was left for 30 min, and the absorbance was measured using a spectrophotometer (Shimadzu UV-1800, Japan) at an absorbance of 370 nm. To create a calibration curve, quercetin is used. The content of total flavonoids in the ethanol extract was expressed as mg quercetin/gm.

### Total tannin content (TTC)

TTC analysis was based on the procedure performed by Kuentzel [19]. The sample weighed 1 gm, which was then dissolved in 100 ml of distilled water. After that, it was extracted by ultrasonics for 15 min at room temperature. The precipitate was separated by centrifugation at 3,000 rpm for 25 min; then, the solution was taken. Take 1 ml of solution and dilute it with 10 ml of distilled water. After that, it was read at a wavelength of 278 nm using a spectrophotometer (Shimadzu UV-1800, Japan).

### Total antioxidant activity (TAA)

TAA analysis was performed by Melia et al. [20] in 375 μl ethanol (99%), and 125 μl DPPH solution (0.02% in ethanol), and 500 μl sample volume was added as a source of free radicals at different concentrations (25 μg/ml, 50 μg/ml, 75 μg/ml, 100 μg/ml, and 125 μg/ml). After 30 min at room temperature, the absorbance of the solution was measured at a wavelength of 517 nm using a spectrophotometer (Shimadzu UV VIS-1800, Japan).

### Data analysis

The obtained data was analyzed using analysis of variance. Continue with Duncan’s multiple range test if the results are significantly different.

## Results and Discussion

In this study, we present the effect of two types of solvents (ordinary distilled water and distilled water heated at 100°C) five different times (15, 30, 45, 60, and 75 min) in the mangosteen leaf extraction process as a feed additive candidate for poultry.

### TPC

The results of the statistical analysis showed that there was a highly significant interaction (*p* < 0.01) between the type of solvent and extraction time for the TPC of mangosteen leaf extract (Table 1). The results of this study showed that the highest TPC (81.03%) was found in ordinary distilled water, with an extraction time of 45 min. This study’s results indicate an increase in TPC in normal distilled water and a longer extraction time. Still, after reaching the optimum time, the additional extraction time can decrease the TPC. In contrast to distilled water heated at 100°C, the highest TPC (34.03%) was found in the extraction time of 30 min, but this figure was much lower than the optimal time with ordinary distilled water. TPC is easily damaged by the heating process and a longer extraction time; in the A2 treatment, the solvent-distilled water used has been preheated to a temperature of 100°C. Heat can cause changes in physicochemical properties and the evaporation of some phenolic compounds until decomposition. This is in line with that reported by Thoo et al. [21]; the longer extraction time reduced the phenolic compounds contained in *Morinda citrifolia*. TPC is also influenced by several factors, such as solvent type and extraction time [22]. Mohammedi et al. [23] stated that the best *Bassia muricata* L. phenol content was extracted using water compared to other solvents such as acetone, ethanol, and hexane. In this study, the phenol content of *Bassia muricata* L. was obtained at 100.12–120.94 mg GAE/gm. The solubility of the phenolic is influenced by the nature of the solvent used and its polarity. Extraction with water solvents also provides advantages such as low cost and non-toxicity compared to other solvents.

According to earlier studies, phenolic compounds, green antimicrobials [24,25], and antioxidants [26,27] could be utilized as natural feed additives in poultry to encourage growth [26,28]. Because of their antioxidant and anti-inflammatory properties, phenolic compounds promote growth by increasing digestive enzyme secretion, decreasing the number of pathogenic bacteria in the digestive tract, or modulating intestinal morphology [29].

### TFC

The interaction between solvent type and extraction time had a significant (*p* < 0.01) effect on the TFC of mangosteen leaf extract (Table 2). TFC mangosteen leaf extract with ordinary distilled water and 45 min (A1B3) gave the best results, namely: 11.07%. This study’s results indicated increased TFC in ordinary distilled water and a longer extraction time. Still, after reaching the optimum time (45 min), the TFC decreased (11.07%–0.66%). Different results were obtained when distilled water was heated to 100°C and longer extraction times caused TFC to be damaged or absent. Flavonoids are phenolic compounds that have conjugated aromatic systems and glycosidic bonds with sugar molecules. Conjugated aromatic systems and glycosidic adhesives will be easily damaged at high temperatures [22]. Different results were reported by Razila [30], indicating that the best conditions to produce TPC of mangosteen peel extract using an ethanol solvent were at 50°C with an extraction time of 59 min. The TPC obtained was 7.78 mg QE/gm. Extraction time and temperature are the most significant factors for flavonoid activity.

**Table 1.** Effect of solvent type and extraction time on the total phenol content of mangosteen leaves (%).

Solvent (A)	Time (B)					Average
	B1	B2	B3	B4	B5	
A1	71.46 ± 1.20^b^	59.98 ± 0.72^c^	81.03 ± 0.54^a^	35.15 ± 0.98^c^	34.26 ± 0.09^c^	56.37 ± 19.58
A2	31.81 ± 1.30^d^	34.03 ± 2.43^c^	31.77 ± 0.59^d^	30.75 ± 1.28^d^	26.97 ± 0.40^e^	31.07 ± 2.67
Average	51.63 ± 21.74	47.00 ± 14.30	56.40 ± 26.98	32.95 ± 2.62	30.62 ± 4.00	

Means in the same variable with different superscripts differ significantly (*p* < 0.01).

A1 = ordinary distilled water; A2 = distilled water heated at 100°C; B1 = 15 min; B2 = 30 min; B3 = 45 min; B4 = 60 min; B5 = 75 min.

Flavonoids benefit the gastrointestinal tract, cardiovascular system, immune system, modulation of lipid metabolism, the release of insulin hormones, and antioxidant activity in broilers [31–34] In the meantime, flavonoids in laying hens can alter the fatty acid profile and lower the cholesterol content of eggs, improving their nutritional quality [35,36].

### TTC

This research showed that there was a highly significant interaction (*p* < 0.01) between the type of solvent and the extraction time for the TTC of mangosteen leaf extract (Table 3). The results of this study showed that the highest TTC was found in ordinary distilled water, with an extraction time of 45 min. This study’s results indicate an increase in TTC in ordinary distilled water and a longer extraction time. Still, after reaching the optimum time, the additional extraction time can decrease the TTC. In contrast to the distilled water, which was heated to 100°C, TTC is damaged or absent after 15–75 min of extraction time. It is suspected that high temperatures can trigger chemical degradation in tannins. Tannins are susceptible to degradation by heat, which can cause changes in their chemical structure and ultimately reduce their tannin content. These results are by Sekarsari et al. [22] reported that the optimum time and temperature for extracting tannins from *Psidium guajava* leaves was 45°C for 20 min; the higher the temperature and time used in the extraction process, the lower the tannin content. Arina and Harisun [37] reported that extraction temperature affects the tannin content of *Quercus infectoria*. At a minimum temperature of 75°C, a tannin amount of 2232.82 mg/gm was obtained, while at 100°C, the tannin content decreased to 2150.74 mg/gm. According to Simamora et al. [38] the distilled water solvent can still maintain tannin levels in the extraction process of *Protium javanicum* *burm. f.* leaves, namely 2.81 ± 0.26 mg TAE/gm.

Feed supplementation containing tannin in poultry can have both positive and negative effects. Feeding low amounts of tannin in the diet has no adverse effects on amino acid digestibility, growth rate, or carcass [39,40]. However, the high amounts of tannin in the ratio can interfere with amino acid digestibility, reduce performance (body weight gain and FCR), and affect lymphoid organs [41].

### Total antioxidant activity

This study showed that the interaction between the type of solvent and extraction time, which was significantly different (*p* < 0.01), reduced the TAA of mangosteen leaves (Table 4). The A1B2 treatment gave the highest TAA of 87.75% compared to the other treatments. The TAA of distilled water heated at 100°C was the lowest and significantly different from the others. This is by Butsat and Siriamornpun [42], who stated that TAA was affected by solvent and extraction time. In this study, the hot distilled water solvent at the beginning of the extraction had a temperature of 100°C; this high temperature was thought to damage the secondary metabolites of the material. The TAA of mangosteen leaf extract increases linearly with increasing antioxidant compounds such as TPC, TFC, and TTC. Still, after reaching optimum conditions (time), the antioxidant activity decreases linearly according to the decrease in these compounds. Similar results were also found by Muhamad et al. [43], who found that ordinary distilled water is more effective in producing antioxidant content in *Cucumis melo*. According to Sekarsari et al. [22], the optimum extraction time for antioxidants in *P. guajava* leaves is 20–30 min. This is the research result obtained: the highest antioxidant content was found in extraction for 30–45 min. Arina and Harisun [37] also reported that extraction temperature affects the antioxidant activity of *Q. infectoria*. At a minimum temperature of 75°C, the antioxidant activity was 93.42%, while at 100°C, the antioxidant activity decreased to 90.76%.

**Table 2.** Effect of solvent type and extraction time on the TFC of mangosteen leaves (%).

Solvent (A)	Time (B)					Average
	B1	B2	B3	B4	B5	
A1	8.97 ± 0.01^b^	8.11 ± 0.20^c^	11.07 ± 0.40^a^	2.19 ± 0.86^d^	0.66 ± 0.25	6.20 ± 4.20
A2	0.00 ± 0.00^f^	0.00 ± 0.00^f^	0.00 ± 0.00^f^	0.00 ± 0.00^f^	0.00 ± 0.00^f^	0.00 ± 0.00
Average	4.49 ± 4.91	4.05 ± 4.44	5.53 ± 6.07	1.09 ± 1.31	0.33 ± 0.39	

Means in the same variable with different superscripts differ significantly (*p* < 0.01).

A1 = ordinary distilled water; A2 = distilled water heated at 100°C; B1 = 15 min; B2 = 30 min; B3 = 45 min; B4 = 60 min; B5 = 75 min.

**Table 3.** Effect of solvent type and extraction time on the total tannin content of mangosteen leaves (%).

Solvent (A)	Time (B)					Average
	B1	B2	B3	B4	B5	
A1	0.83 ± 0.02^b^	0.71 ± 0.01^c^	1.01 ± 0.02^a^	0.00 ± 0.00^d^	0.00 ± 0.00^d^	0.51 ± 0.44
A2	0.00 ± 0.00^d^	0.00 ± 0.00^d^	0.00 ± 0.00^d^	0.00 ± 0.00^d^	0.00 ± 0.00^d^	0.00 ± 0.00
Average	0.42 ± 0.45	0.35 ± 0.39	0.51 ± 0.55	0.00 ± 0.00	0.00 ± 0.00	

Means in the same variable with different superscripts differ significantly (*p* < 0.01).

A1 = ordinary distilled water; A2 = distilled water heated at 100°C; B1 = 15 min; B2 = 30 min; B3 = 45 min; B4 = 60 min; B5 = 75 min.

**Table 4.** Effect of solvent type and extraction time on the TAA of mangosteen leaves (%).

Solvent (A)	Time (B)					Average
	B1	B2	B3	B4	B5	
A1	69.85 ± 0.67^c^	87.75 ± 2.47^a^	77.61 ± 1.48^b^	35.67 ± 0.65^f^	28.76 ± 2.13^g^	59.93 ± 24.29
A2	40.99 ± 2.24^d^	43.88 ± 1.24^de^	38.80 ± 0.74^ef^	29.69 ± 5.40^g^	14.85 ± 2.70^h^	33.64 ± 11.18
Average	55.42 ± 15.87	65.81 ± 24.09	58.20 ± 21.28	32.68 ± 4.75	21.80 ± 7.93	

Means in the same variable with different superscripts differ significantly (*p* < 0.01).

A1 = ordinary distilled water; A2 = distilled water heated at 100°C; B1 = 15 min; B2 = 30 min; B3 = 45 min; B4 = 60 min; B5 = 75 min.

In poultry production, antioxidants are essential. Natural antioxidants can improve product quality, lower oxidative stress, and enhance general health [11,31,44–46]. It has been demonstrated that natural antioxidants can either entirely or partially replace synthetic antioxidant vitamins in lowering oxidative stress in chickens [11].

The weaknesses of our research are: (i) This study is at an early stage in determining the effective solvent and time to produce phytochemical compounds. (ii) Further research on the in vitro test of extraction mangostenn leaf against several pathogenic bacteria (*Salmonella enterica*, *Escherichia coli*, etc.) in the poultry gastrointestinal tract as antibacterial is needed, and (iii) further research on *in vivo* tests on poultry (especially broilers) is needed.

## Conclusion

The best solvent and time for mangosteen leaf extract to produce secondary metabolites, which can be candidates for feed additives in poultry, is ordinary distilled water for 45 min. In this research, the phenol content was 81.03%, flavonoids 11.07%, tannins 1.01%, and antioxidants 77.61%.

## List of Abbreviations

AGP, antibiotic growth promoters; GAE, gallic acid equivalent; TFC, total flavonoid content; TPC, total phenolic content; TTC, Total tannin content; TAA, Total antioxidant activity.

## References

[1] Ramadhanti AR, Puspita NOJ, Refalta CF, Kurnianto H, Saragih HTS (2021). Performance of male layer fed ration containing green algae (*Spirogyra jaoensis*) extract. Trop Anim Sci J.

[2] Irawan A, Hidayat C, Sholikin MM, Harahap RP, Rusli RK, Solfaine R (2021). Propolis supplementation on broiler chicken performances, nutrient digestibility, and carcass characteristics: a meta-analysis. Trop Anim Sci J.

[3] Zhang LY, Peng QY, Liu YR, Ma QG, Zhang JY, Guo YP (2021). Effects of oregano essential oil as an antibiotic growth promoter alternative on growth performance, antioxidant status, and intestinal health of broilers. Poult Sci.

[4] Maesaroh U, Dono ND, Zupriza Z (2022). Performance, microbial populations, and jejunal morphology of broilers supplemented with nano-encapsulated graviola leaf extract. Trop Anim Sci J.

[5] Dosoky WM, Zeweil HS, Ahmed MH, Zahran SM, Shaalan MM, Abdelsalam NR (2021). Impacts of onion and cinnamon supplementation as natural additives on the performance, egg quality, and immunity in laying Japanese quail. Poult Sci.

[6] Albuquerque BR, Dias MI, Pinela J, Calhelha RC, Pires TCSP, Alves MJ (2023). Insights into the chemical composition and *in vitro* bioactive properties of mangosteen (*Garcinia mangostana* L.) pericarp. Foods.

[7] Tran VA, Thi Vo TT, Nguyen MNT, Duy Dat N, Doan VD, Nguyen TQ (2021). Novel α -mangostin derivatives from mangosteen (*Garcinia mangostana* L.) peel extract with antioxidant and anticancer potential. J Chem.

[8] Nseme YDM, Mandeng KFP, Mounpou J, Djuikoo ILN, Nguedjo MW, Baleba RMM (2022). Bioactive compounds from mangosteen fruit peels (*Garcinia mangostana* L.) and assessment of their antioxidant potential. Microbiol Res J Int.

[9] Herawati O, Untari T, Anggita M, Artanto S (2020). Effect of mangosteen (*Garcinia mangostana* L.) peel extract as an antibiotic growth promoter on growth performance and antibiotic resistance in broilers. Vet World.

[10] Boontiam W, Kumari P (2019). Effect of mangosteen (*Garcinia mangostana*) pericarp extract in reducing the heat stress of laying quails. Adv Anim Vet Sci.

[11] Rusli R, Mutia R, Wiryawan K, Toharmat T, Jakaria J (2015). Effect of mangosteen pericarp meal and vitamin E supplements on the performance, blood profiles, antioxidant enzyme and HSP 70 gene expression of laying hens in tropical environment. Int J Poult Sci.

[12] Rusli RK, Wiryawan KG, Toharmat T, Jakaria, Mutia R (2015). Supplementation of mangosteen pericarp meal and vitamin E on egg quality and blood profile of laying hens. Media Peternakan Fakultas Peternakan Institut Pertanian Bogor.

[13] Suhartati R, Apriyani F, Khusnul, Virgianti DP, Fathurohman M (2019). Antimicrobial activity test of mangosteen leaves ethanol extract (*Garcinia mangostana* Linn) against *Pseudomonas aeruginosa *bacteria. J Phys Conf Ser.

[14] Diniatik A K, Siswanto A, Doti DM (2020). Antioxidant activities and identification of compounds in ethyl acetate fraction, dichloromethane fraction and ethanol extract of *Garcinia mangostana* L. leaves. Int J Pharm Res.

[15] Palakawong C, Delaquis P (2018). Mangosteen processing: A review. J Food Process Preserv..

[16] Yassin MT, Al-Askar AA, Maniah K, Al-Otibi FO (2022). Green synthesis of zinc oxide nanocrystals utilizing *Origanum majorana* leaf extract and their synergistic patterns with colistin against multidrug-resistant bacterial strains. Crystals (Basel).

[17] Calvindi J, Syukur M, Nurcholis W (2020). Investigation of biochemical characters and antioxidant properties of different winged bean (*Psophocarpus tetragonolobus*) genotypes grown in Indonesia. Biodiversitas.

[18] Chang C, Yang M, Wen H, Chern J (2002). Estimation of total flavonoid content in propolis by two complementary colorimetric methods. J Food Drug Anal.

[19] Kuentzel A (1955). Gerbereichemisches taschenbuch. the Verlag Theodor Steinkopff.

[20] Melia S, Juliyarsi I, Kurnia YF (2022). Physicochemical properties, sensory characteristics, and antioxidant activity of the goat milk yogurt probiotic *Pediococcus acidilactici* BK01 on the addition of red ginger (*Zingiber officinale* var. rubrum rhizoma). Vet World.

[21] Thoo YY, Ho SK, Liang JY, Ho CW, Tan CP (2010). Effects of binary solvent extraction system, extraction time and extraction temperature on phenolic antioxidants and antioxidant capacity from mengkudu (*Morinda citrifolia*). Food Chem.

[22] Sekarsari S, Widarta I, Jambe AA (2019). The influence of time and temperature with ultrasonic waves on antioxidant activity of extracts guajava leaves (*Psidium guajava* L.). J Ilmu Dan Teknologi Pangan.

[23] Mohammedi H, Idjeri-Mecherara S, Menaceur F, Hassani A (2019). The effect of solvents and extraction procedure on the recovery of phenolic compounds and the antioxidant capacity of Algerian *Bassia muricata* L. extracts. Chem J Moldova.

[24] Mehri M, Sabaghi V, Bagherzadeh-Kasmani F (2015). *Mentha piperita* (peppermint) in growing Japanese quails diet: performance, carcass attributes, morphology and microbial populations of intestine. Anim Feed Sci Technol.

[25] Salaheen S, Tabashsum Z, Gaspard S, Dattilio A, Tran TH, Biswas D (2018). Reduced *Campylobacter jejuni* colonization in poultry gut with bioactive phenolics. Food Control.

[26] Starčević K, Krstulović L, Brozić D, Maurić M, Stojević Z, Mikulec Ž (2015). Production performance, meat composition and oxidative susceptibility in broiler chicken fed with different phenolic compounds. J Sci Food Agric.

[27] He S, Li S, Arowolo MA, Yu Q, Chen F, Hu R (2019). Effect of resveratrol on growth performance, rectal temperature and serum parameters of yellow-feather broilers under heat stress. Anim Sci J.

[28] Chen Y, Chen H, Li W, Miao J, Chen N, Shao X (2018). Polyphenols in eucalyptus leaves improved the egg and meat qualities and protected against ethanol-induced oxidative damage in laying hens. J Anim Physiol Anim Nutr (Berl).

[29] Mahfuz S, Shang Q, Piao X (2021). Phenolic compounds as natural feed additives in poultry and swine diets: a review. J Anim Sci Biotechnol.

[30] Razila A, Ramli SS (2021). Optimisation of mangosteen peel extracts (*Garcinia mangostana* L.) on total flavonoid content using response surface methodology (RSM) and its antioxidant activities.

[31] Prihambodo TR, Sholikin MM, Qomariyah N, Jayanegara A, Batubara I, Utomo DB (2021). Effects of dietary flavonoids on performance, blood constituents, carcass composition and small intestinal morphology of broilers: a meta-analysis. Anim Biosci.

[32] Zhou Y, Mao S, Zhou M (2019). Effect of the flavonoid baicalein as a feed additive on the growth performance, immunity, and antioxidant capacity of broiler chickens. Poult Sci.

[33] Parmar A, Patel V, Patel J, Usadadia S, Rathwa S, Prajapati D (2019). Quercetin, a health promising phytoadditive for poultry production: trends & advances. Pharm Innov J.

[34] Zhang S, Kim IH (2020). Effect of quercetin (flavonoid) supplementation on growth performance, meat stability, and immunological response in broiler chickens. Livest Sci.

[35] Kamboh AA, Leghari RA, Khan MA, Kaka U, Naseer M, Sazili AQ (2019). Flavonoids supplementation-an ideal approach to improve quality of poultry products. Worlds Poult Sci J.

[36] Tan Z, Halter B, Liu D, Gilbert ER, Cline MA (2022). Dietary flavonoids as modulators of lipid metabolism in poultry. Front Physiol.

[37] Arina MZ, Harisun Y (2019). Effect of extraction temperatures on tannin content and antioxidant activity of *Quercus infectoria* (Manjakani). Biocatal Agric Biotechnol.

[38] Simamora ACY, Yusasrini NLA, Putra INK (2021). Effect of different solvent on the antioxidant activity of Tenggulun Leaves extract (*Protium javanicum Burm. F*) with the maceration method. Itepa.

[39] Kopmels FC, Smit MN, Cho M, He L, Beltranena E (2020). Effect of feeding 3 zero-tannin faba bean cultivars at 3 increasing inclusion levels on growth performance, carcass traits, and yield of saleable cuts of broiler chickens. Poult Sci.

[40] Puntigam R, Brugger D, Slama J, Inhuber V, Boden B, Krammer V (2020). The effects of a partial or total replacement of ground corn with ground and whole-grain low-tannin sorghum (*Sorghum bicolor* (L.) Moench) on zootechnical performance, carcass traits and apparent ileal amino acid digestibility of broiler chickens. Livest Sci.

[41] Hidayat C, Irawan A, Jayanegara A, Sholikin MM, Prihambodo TR, Yanza YR (2021). Effect of dietary tannins on the performance, lymphoid organ weight, and amino acid ileal digestibility of broiler chickens: a meta-analysis. Vet World.

[42] Butsat S, Siriamornpun S (2016). Effect of solvent types and extraction times on phenolic and flavonoid contents and antioxidant activity in leaf extracts of amomum Chinense C. Int Food Res J.

[43] Muhamad N, Sahadan W, Hoon H (2018). Effect of drying temperatures and extraction solvents on total phenolic, flavonoid contents and antioxidant properties of immature Manis Terengganu Melon (*Cucumis melo*). J Agrobiotech.

[44] Hidayat C (2018). Synthesis of zinc nanoparticles using plant extract for broiler’s feed additive. Indonesian Bull Anim Vet Sci.

[45] Hidayat C, Sumiati S, Jayanegara A, Wina E (2021). Supplementation of dietary nano Zn-phytogenic on performance, antioxidant activity, and population of intestinal pathogenic bacteria in broiler chickens. Trop Anim Sci J.

[46] Hidayat C, Sumiati, Jayanegara A, Wina E (2020). Effect of zinc on the immune response and production performance of broilers: a meta-analysis. Asian-Aust J Anim Sci.

